# Right to Appeal, Non-Treatment, and Violence Among Forensic and Civil Inpatients Awaiting Incapacity Appeal Decisions in Ontario

**DOI:** 10.3389/fpsyt.2019.00752

**Published:** 2019-11-08

**Authors:** Radovan Radisic, Nathan J. Kolla

**Affiliations:** ^1^Forensic Psychiatry Service, Centre for Addiction and Mental Health (CAMH), Toronto, ON, Canada; ^2^Violence Prevention Neurobiological Research Unit, CAMH, Toronto, ON, Canada; ^3^Department of Psychiatry, University of Toronto, Toronto, ON, Canada; ^4^Centre for Criminology & Sociolegal Studies, University of Toronto, Toronto, ON, Canada; ^5^Waypoint Centre for Mental Health Care, Penetanguishene, ON, Canada

**Keywords:** forensic psychiatry, incapacity, violence, mental health legislation, treatment delay

## Abstract

**Background:** Mental health legislation in Ontario, Canada, permits inpatients to refuse treatment while appealing their incapacity finding to the Consent and Capacity Board (CCB). Lack of treatment during this period poses safety concerns, as inpatients who remain untreated are at higher risk of engaging in violent behavior. The present study explored the relationship between non-treatment and violence among forensic and civil inpatients awaiting their CCB hearing at the largest psychiatric hospital in Canada.

**Methods:** We investigated the electronic health records of 285 inpatients whose CCB applications were heard between 2014 and 2016 to better understand violent outcomes among inpatients and determine whether application timelines differed between forensic and civil inpatients.

**Results:** Three key findings were observed. First, forensic inpatients had more episodes of violence requiring seclusion and restraint during the application timeline compared with civil inpatients. Second, forensic inpatients waited longer than civil inpatients for their appeal to be heard at the CCB. Finally, unwillingness to accept PRN medications and comorbid psychiatric conditions were potent risk factors for violence among all inpatients during the appeals process.

**Conclusions:** Compared with civil inpatients, forensic inpatients waited longer for CCB appeals. They also scored higher on one measure of violent behavior. These findings provide context for the ongoing challenge of clinicians tasked with providing care for inpatients appealing findings of incapacity under mental health legislation in Ontario. We argue for a more streamlined approach to processing appeals for both forensic and civil patients. Better standardization or even revision of current mental health legislation may help eliminate clinical disparities between patient groups.

## Introduction

The interaction between the criminal justice system and psychiatric services has demonstrated a tenuous relationship between balancing individual rights and public safety. In Ontario, Canada, according to s.18(1) of the *Health Care Consent Act, 1996*, no treatment may be administered to involuntarily detained inpatients if they appeal the finding of incapacity to consent to treatment before the Consent and Capacity Board (CCB), an independent tribunal established to facilitate hearings for review under the *Health Care Consent Act, 1996* and the *Mental Health Act, 1990*. The CCB then decides whether individuals are capable of making their own treatment decisions. Despite growing reliance on legal mechanisms to supplement psychiatric treatment plans, few studies have analyzed the emergence of violence among forensic and civil inpatients during the appeals process when inpatients remain untreated with psychotropic medications. Both forensic and civil inpatients are vulnerable to violent behavior during the appeals process due to lack of treatment that typically reduces violence (1). Therefore, our research was guided by the following aims: First, we explored a range of demographic and clinical variables to study the frequency of violence during the appeals process among forensic and civil inpatients admitted under involuntary psychiatric care in Ontario who were awaiting their CCB hearing. Second, we explored the length of appeal in both forensic and civil patients to understand whether patients in one group waited significantly longer for the resolution of their appeals, thus prolonging the period of non-treatment for patients in that group. Third, we ascertained whether patients with comorbid conditions, which we interpreted as a measure of clinical severity, waited longer for their CCB appeal.

Much of the literature describing the relationship between mental health and the law has primarily provided an analysis of the historical evolution of legal and psychiatric principles infused as core tenets guiding the objectives of contemporary mental health legislation (2–9). Hartford et al. (3) argue that a “rights revolution” has continuously gained prominence in Canadian mental health law reform, which has afforded greater freedoms to individual inpatients, such as their ability to participate and consent to psychiatric treatment. The impact of this rights-based movement on mental health legislation has been analyzed by other scholars. For example, O’Reilly et al. (6) examined the various types of legal safeguards and minimum protections for inpatients and investigated the expansion of greater autonomy and access to courts and administrative tribunals, which are the venues for legal challenges brought under mental health legislation. Although a delay to treatment strives to ensure that inpatients’ rights are not compromised in clinical settings, other authors have studied the detrimental impacts of non-treatment on inpatients. For instance, numerous studies have shown that non-treatment is associated with longer rates of hospitalization and greater frequency of relapse (10–13).

Among patients with schizophrenia, non-adherence to treatment is commonly associated with a lack of awareness or refusal to acknowledge illness (14, 15). Refusal of PRN medications, agents that are not normally scheduled medications but are used to acutely subdue psychiatric symptoms, has been associated with a greater number of suicide attempts, longer hospitalizations, and higher rates of relapse (16). The effect of symptom burden, including positive and negative symptoms in schizophrenia, has also been shown to make treatment refusal more likely (17–20), and treatment refusal has been linked to an increase in patient violence (21). On the other hand, a willingness to accept medication and engage in treatment programs is typically associated with positive results for the patients (22–28). Finally, Greenberg (5) argues that the automatic right to appeal to the CCB in Ontario, rather than a right to review in most other Canadian jurisdictions that would permit concurrent treatment during the review process, likely produces negative clinical effects on individual inpatients with major mental illness, as procedural delays may prolong necessary treatment.

First, we hypothesized that forensic inpatients would display higher rates of violence than civil inpatients during the wait to the CCB hearing, given that the majority of the forensic inpatients would have been hospitalized for commission of a violent act. Second, we further hypothesized that forensic inpatients would wait longer than civil inpatients for their CCB hearing, due to legal complexities and greater administrative burdens associated with forensic inpatients. Third, we hypothesized that patients with comorbid psychiatric conditions would wait the longest for their appeals, as this presentation may influence the complexity of the appeals process. These hypotheses were structured to answer the overarching questions of whether the appeals process operated differently for forensic versus civil inpatients and whether clinical data could predict violent behavior that emerged while waiting for the appeal to be heard.

## Methods

### Design

This investigation is a retrospective cross sectional study that examined the electronic health records (EHRs) of 285 involuntary inpatients at the Centre for Addiction and Mental Health (CAMH) in Toronto, Ontario, who applied for an appeal of their finding of incapacity to the CCB. Study variables included age, sex, violence, length of CCB appeal, patient type (forensic versus civil), diagnosis, unwillingness to take PRN medication, and comorbid psychiatric conditions.

### Variables

The independent variable was type of patient, for example, civil or forensic. CAMH is a general and forensic hospital that treats forensic patients detained under Ontario Review Board dispositions and civil psychiatric patients who may be admitted for voluntary or involuntary hospitalization. The dependent variable was patient violence. Confounding variables included patient age, sex, psychiatric diagnoses, length of CCB application, unwillingness to take PRN medications, and comorbid psychiatric disorders.

#### Violence

Each episode of violence gleaned from the inpatients’ EHR was recorded. We defined a violent episode as a recorded and identified instance where an inpatient exercised a visible form of physical aggression, whether or not that aggression was inflicted upon another individual. For example, physical aggression could be directed toward people or objects. For the purposes of this study, episodes of violence were divided into three categories: 1) physical aggression not requiring a code white response or restraint/seclusion tactics; 2) physical aggression triggering a code white alarm (e.g., episodes where hospital emergency protocols are exercised to defuse violent inpatients); and, 3) physical aggression requiring the use of restraint/seclusion to subdue the inpatient (e.g., application of restraint/seclusion tactics by hospital staff in response to intractable violence). The use of restraint/seclusion identified the most intense form of violence, as all inpatients displaying this behavior also necessitated the use of a code white alarm (violence/behavioral situation). For some inpatients, more than one category of violence was recorded during one event, and some may have occurred simultaneously, depending on the specific circumstances. However, only the highest level of aggression was considered (e.g., inpatients were not recorded as having engaged in two acts of physical aggression if both restraints and a code white were employed). Violent behaviors were recorded using both dichotomous and continuous variables. For dichotomous variables, inpatients were categorized as violent if they had demonstrated at least one or more episodes of violence during their application timeline. The total number of discrete violent episodes per inpatient was also recorded, although, as noted above, multiple forms of violence that had occurred contemporaneously were treated as one event.

#### Length of CCB Appeal

The number of days that had elapsed from the day the CCB application was filed to the date a decision was rendered was recorded. This variable reflects the minimum number of days that inpatients were untreated with psychotropic medications.

#### Diagnoses

We recorded diagnoses made by clinicians at the time of the CCB application. One group was created that included all patients with either schizophrenia or schizoaffective disorder, another group included individuals with a psychotic disorder other than schizophrenia or schizoaffective disorder, a third group included all patients with a substance use disorder, and a final group comprised patients with personality disorders. We created another variable (comorbid conditions) that included patients with two or more of the above diagnoses. It was coded dichotomously (e.g., yes/no).

#### PRN Medication

We recorded instances where PRN medication was offered to inpatients who displayed signs of violence. Unwillingness to accept PRN medication was measured as a dichotomous variable and indicated whether inpatients had refused or accepted PRN medications during the entire application timeline period. For example, if inpatients refused PRN medication after having instigated a violent episode, they would be recorded as having been unwilling to accept PRN medications, regardless of their willingness to accept PRN medications on all other occasions. PRN medications included antipsychotics and benzodiazepines.

### Study Setting

CAMH is a stand-alone speciality psychiatric hospital located in Toronto, Ontario, and the largest mental health and addictions hospital in Canada.

### Sample

All civil and forensic inpatients who had applied for CCB relief (*n* = 285) between January 2014 and April 2016 were included in this analysis. A change in the EHR system in 2016 made it difficult to collate data from April 2016 and beyond. Therefore, we began reviewing charts in 2014 to capture at least two years’ worth of data. Both forensic inpatients (*n* = 31) and civil inpatients (*n* = 254) had hearings from CCB applications that were lodged and heard during this period.

A sample of 285 subjects provided 80% power to detect an odds ratio of 1.96 at a significance level of 0.05, considering a predictor with two levels and equal sample size in each level and also assuming a baseline prevalence of violent incidents of 43%. The detectable odds ratio increased to 2.35 when the binary predictor had categories split at an 80%/20% ratio. Considering a baseline proportion of 30%, the odds ratio of 1.96 is equivalent to a change in prevalence to 46% and an odds ratio of 2.35 to a change in prevalence from 30% to 50%. This power calculation was conducted using G*Power 3.1.9.2 (29).

### Process

The data for this study were retrieved from the EHR database at CAMH. CAMH uses an EHR management system to record all patient encounters, which includes notes written by physicians and allied healthcare staff as well as legal forms. Other information, such as inpatient admission/discharge dates and applications and timelines to the CCB (and their outcomes), are also contained within the EHR. Consistent with clinical practice, staff documented inpatients’ behavior on a daily basis, including any episodes of violence or aggression.

### Ethics

All study components were approved by the CAMH Research Ethics Board.

### Statistics

Chi-square tests and independent samples *t*-tests were employed to analyze clinical and demographic variables. To test which clinical and demographic variables predicted patient violence, we fit both unadjusted and adjusted logistic regression models using backward deselection. For a backward deselection logistic regression procedure, the least significant predictor is removed from the model until no predictor is found to be significant at a significance level of 0.2. The procedure begins with all predictors in the model at the initial step. Then, predictors are removed based on the probability of the likelihood-ratio statistic according to maximum partial likelihood estimates (30).

We included the following variables as independent predictors in the unadjusted and adjusted logistic regression: age (years), sex (male/female), patient type (forensic/civil), schizophrenia/schizoaffective disorder (yes/no), other psychotic disorder (yes/no), substance use disorder (yes/no), personality disorder (yes/no), comorbid conditions (yes/no), appeal timeline (number of days), and unwillingness to take PRN medication (yes/no).

Age and sex were selected, as younger and male patients typically endorse higher rates of violence (31). Furthermore, clinical diagnoses and comorbidity are relevant to our understanding of whether violence is associated with specific psychiatric conditions or more comorbidity (32). Non-adherance to psychiatric medications has also been linked to violent outcomes (26, 16).

## Results

### Sample Description

From the complete sample of forensic and civil inpatients (*n* = 285), 66% were male (*n* = 188) and 34% were female (*n* = 97). The average age was 40.0 ± 15.8 years. There was no significant difference in age between the forensic and civil inpatients (forensic: 43.3 ± 13.6 years; civil: 39.6 years ± 16.0 years; *t* = 1.2, *p* = .22). However, there was a significant difference in the distribution of male and female inpatients according to forensic/civil inpatient classification (forensic inpatients: male 90.3%/female 9.7%; civil inpatients: male 63.0%/female 37.0%; (χ(1) = 9.2, *p* = .002). Forensic patients (*n* = 31, 11%) and civil inpatients (*n* = 254, 89%) comprised the total sample of 285 patients.

### Diagnoses

59.3% (*n* = 169) of the patients were diagnosed with schizophrenia/schizoaffective disorder, 31.2% (*n* = 89) were diagnosed with a psychotic disorder other than schizophrenia/schizoaffective disorder, 15.1% (*n* = 43) were diagnosed with a substance use disorder, and 16.1% (*n* = 46) were diagnosed with a personality disorder. 22.5% (*n* = 64) of patients had comorbid psychiatric conditions.

### Violence During the Appeals Process

There was a trend relationship toward forensic inpatients engaging in more violent episodes during the CCB application timeline (1.6 ± 2.4 violent episodes in the forensic group versus 1.0 ± 1.7 episodes in the civil group; *t* = 1.7, *p* = .090). Among the different classifications of violence (e.g., physical aggression, code white, and restraint/seclusion), there were no differences between forensic and civil inpatients during the application timeline, save for the use of restraints/seclusion, where the number of incidents was greater in the forensic group (forensic: 0.5 ± 0.9 episodes, civil: 0.2 ± 0.6 episodes; *t* = 2.42, *p* = .016). However, there was no difference in the proportion of forensic inpatients (11.5%) versus civil inpatients (10.4%) classified as violent (χ^2^(1) = 0.79, *p* = 0.78) during the application timeline.

### Length of CCB Appeal

The mean length of time to the CCB hearing was longer for forensic inpatients (28.9 ± 64.2 days) than civil inpatients (13.3 ± 11.2 days; *t* = 3.5, *p* < .001). There was a trend relationship for patients with psychiatric comorbidities waiting longer for their CCB appeal than patients without comorbidities (19.8 ± 45.9 days versus 13.6 ± 11.2 days; *t* = 1.8, *p* = 0.069).

### Unwillingness to Accept PRN Medication

As expected, among all inpatients who accepted PRN medication when offered, there were fewer violent episodes (0.45 ± 1.1 episodes) compared with inpatients who did not accept PRN medication (2.1 ± 2.1 violent episodes; *t* = −8.5; *p* < .001). However, there was no difference in the proportion of inpatients accepting PRN medication between forensic and civil inpatients (χ^2^(1) = 0.10, *p* = 0.75).

### Predictors of Violence

In the unadjusted logistic regressions, unwillingness to accept PRN medications (β = 2.2; *p* < 0.001; 95% confidence interval = 0.06–0.19), comorbid psychiatric conditions (β = 0.79; *p* = 0.006; 95% confidence interval = 1.2–3.9), and personality disorder diagnosis (β = 0.88; *p* = 0.008; 95% confidence interval = 1.3–4.6) each predicted violent behavior during the CCB application timeline among all inpatients (**Table 1**). However, for the adjusted logistic regression, only unwillingness to accept PRN medications (β = 2.2; *p* < 0.001; 95% confidence interval = 0.06–0.19) and comorbid psychiatric conditions (β = 1.1; *p* = 0.011; 95% confidence interval = 1.3–7.6) predicted violent behavior during the CCB application timeline for all inpatients (**Table 2**).

**Table 1:** Demographic and Clinical Predictors of Violence (Unadjusted Odds Ratios).

Variable	*B*	S.E.	Wald	Df	Sig.	Exp(*B*)	95% C.I.	95% C.I.
**Age**	–.008	0.008	1.1	1	.31	.99	.98	1.0
**Sex**	.16	.25	.41	1	.52	1.18	.72	1.93
**Schizophrenia/Schizoaffective Disorder Diagnosis**	–.44	.24	3.2	1	.074	.65	.40	1.04
**Psychosis Diagnosis**	–.39	.26	2.3	1	.13	.68	.41	1.12
**Substance Use Disorder Diagnosis**	.18	.33	.28	1	.59	1.19	.62	2.29
**Personality Disorder Diagnosis**	.88	.33	7.0	1	.008	2.4	1.3	4.58
**Patient Type**	.11	.38	.079	1	.78	1.11	.53	2.36
**Comorbid Conditions**	.79	.29	7.41	1	.006	2.19	1.25	3.86
**Length of Time of CCB Appeal**	.002	.005	.11	1	.74	1.0	.99	1.01
**Unwillingness to take PRN Medication**	2.21	.28	62.41	1	0	.11	.064	.190

**Table 2:** Demographic and Clinical Predictors of Violence (Adjusted Odds Ratios).

Variable	*B*	S.E.	Wald	Df	Sig.	Exp(*B*)	95% C.I.	95% C.I.
**Unwillingness to take PRN Medication**	2.21	.29	59.58	1	.000	.11	.064	.19
**Substance Use Disorder Diagnosis**	–.93	.52	3.19	1	.074	.39	.14	1.10
**Comorbid Conditions**	1.15	.45	6.44	1	.011	3.14	1.23	7.61
**Constant**	.88	.23	14.56	1	.000	2.42	—	—

## Discussion

The aim of this study was three-fold. First, we explored whether forensic inpatients would display more violence during the CCB appeals process than civil inpatients. Second, we investigated whether forensic inpatients compared with civil inpatients would wait longer for resolution of the CCB appeals process. Third, we were interested to learn whether patients with greater illness burden, indexed by comorbid psychiatric conditions, waited longer for their CCB appeal to be heard. Several findings emerged. First, we found that forensic inpatients had more episodes of the use of restraints and seclusion during the CCB waiting period compared with civil inpatients. However, there was no difference between forensic and civil inpatients on all other measures of violence. Second, we ascertained that the length of time to appeal was longer for forensic inpatients than civil inpatients. Third, we discovered that the length to appeal was not significantly longer for patients with comorbid psychiatric conditions versus patients with only one psychiatric diagnosis. Fourth, analyses revealed that unwillingness to take PRN medication and the presence of comorbid psychiatric conditions were significant predictors of violent outcomes among all patients.

There may be several explanations to describe our finding that forensic inpatients had a greater number of violent incidents that warranted the use of restraint and seclusion. Typically, forensic inpatients are more likely to be admitted to hospital as a result of violent offending and thus have a greater propensity to engage in violent behavior (33). Some evidence suggests that forensic patients may be more susceptible to violence compared with civil patients when they do not receive psychotropic medication (34). Finally, forensic inpatients waited longer for their CCB appeal to be processed. Therefore, a heightened vulnerability to violence during periods of non-treatment coupled with longer CCB application timelines may have increased the likelihood of violence in this sample. It is also probable that forensic inpatients were more closely observed compared with civil inpatients, making detection of violence among forensic inpatients more likely (35). This finding suggests that clinicians ought to be particularly vigilant for signs and symptoms of increased violence in forensic inpatients during the appeals process. Use of all available pharmacological and non-pharmacological methods should be strongly considered when eruption of violence is suspected. However, on-going risk assessment is still necessary to circumvent imminent violence or aggression, and risk management is then required to prevent violence. Hence, these management strategies should be initially based on the least restrictive options. The use of restrictive policies to contain violence should only be employed when least restrictive options have failed to contain the behavior.

Our second main finding was that the length of time to treatment was longer for forensic inpatients. Lengthier periods of non-treatment pose challenges for both inpatients and clinicians, as inpatients may have greater opportunity to perpetrate violence. We did not have data that could shed light on procedural matters related to CCB hearings, which could potentially outline reasons for the relative delay of forensic inpatients. However, it is likely that hearings involving forensic inpatients are more litigious, given the multitude of legal complexities and higher risk associated with forensic status (35). Future research could be directed to examine factors that influence the CCB application timeline for forensic and civil inpatients.

Finally, results indicated that unwillingness to accept PRN medications and comorbid psychiatric conditions both predicted violent outcomes among the entire sample of inpatients. Consistent with earlier references cited, patients who adhere to medication generally show lower rates of violence compared to patients who are non-adherent (16, 22–28). Although administration of PRN medication does not replicate the treatment plan that would otherwise be prescribed to inpatients based on their diagnosis, use of benzodiazepines and antipsychotics would be expected to reduce agitation and lessen the risk of aggression. Both are commonly used as standing orders for pharmacological regimens. It is also possible that inpatients who were willing to accept PRN medications had greater insight into their condition and were overall less likely to engage in violent behavior. Research elsewhere has also suggested that when more psychiatric comorbidity is present, there is a greater risk of violence (21, 36), which agrees with our finding that inpatients who posed the most clinically complex presentations were more violent.

These observations provide fertile ground for future analyses on how patient violence may correlate with clinical and demographic characteristics during the appeals process in Ontario when patients remain untreated. The current appeal mechanism for patients in Ontario provides a venue for due process and a review of the decision-making by physicians proposing treatment with psychotropic medications. However, our data suggest that some of the most vulnerable patients may be at greatest risk of harm when treatment is delayed. Accordingly, the delays created by the length of time waiting for conclusion of CCB appeals must be examined, as they demonstrate the need to implement a streamlined process for appeal applications that eliminates unreasonable delay caused by administrative aspects of the process. Proposed approaches for eliminating these delays have been raised elsewhere. For example, Solomon et al. (37) analyzed several cases of incapacity challenges and linked them to different areas of mental health legislation in Ontario. They provided several approaches for addressing the issue of delayed treatment for psychiatric patients, including some examples that targeted changes to the mental health legislation itself.

### Limitations

Several study limitations must be noted. First, one of the inherent weaknesses of retrospective studies is their reliance on secondary data sources. Second, inpatients were categorized based on clinical diagnoses and did not undergo standardized testing with validated instruments to obtain diagnoses. As a result, the information available to us may have lacked diagnostic precision. Third, demographic variables were restricted to age and sex, which reduced our ability to provide a more comprehensive analysis on the role of demographic characteristics. Fourth, lack of knowledge about individual clinician’s practices hampered our ability to better understand how certain variables impacted the emergence of violence. For example, clinicians may have had different thresholds for offering PRNs to inpatients. A fifth limitation is that we limited our data collection to two years. As noted above, a new EHR was introduced to the hospital in 2016. At the time that our study was initiated in late 2016, health records services were unable to extract the variables that were required from the new EHR system. Hence, a larger sample size would have increased our power to detect an effect. A sixth limitation is that the prevalence of substance use diagnoses in our sample was relatively low. The lower number was likely due to a combination of factors, including underreporting by patients and physicians only recording the main diagnosis (e.g., schizophrenia). Finally, although our findings show that the length of time to treatment was the longest for forensic inpatients, specific data that might explain procedural delays were unavailable to us. Thus, we may only speculate on reasons for these delays.

### Future Directions

The administration of psychiatric care for involuntarily hospitalized inpatients presents an ongoing challenge for physicians as well as policy-makers. The increasing reliance of the criminal justice system on mental health systems for treatment of individuals in the forensic system makes understanding how violence can vary between inpatient groups paramount. The right of inpatients to appeal their finding of lack of capacity to consent to treatment under the *Mental Health Act, 1990* and the *Health Care Consent Act, 1996* presents an ongoing challenge for clinicians in Ontario to address the effects of untreated mental illness during the appeals process. Effective response to the management of violence must address shortcomings in legislation, institutional practice, as well as treatment methods. Although the current study cannot address all institutional and clinical variables that predispose to violent behavior among forensic and civil inpatients, future research should be aimed at exploring the ways in which the broader contexts of institutional clinical practice and timelines to treatment may influence violence among forensic and civil groups.

## Data Availability Statement

The datasets generated for this study are available on request to the corresponding author.

## Ethics Statement

The studies involving human participants were reviewed and approved by Centre for Addiction and Mental Health Research Ethics Board. Written informed consent for participation was not required for this study in accordance with the national legislation and the institutional requirements.

## Author Contributions

RR was responsible for collecting the data and writing the manuscript. NK was responsible for conceptualizing the study and writing the manuscript.

## Funding

A Canadian Institutes of Health Research Clinician Scientist Phase II Award provided salary support for NK and payment for open access publication fees.

## Conflict of Interest

The authors declare that the research was conducted in the absence of any commercial or financial relationships that could be construed as a potential conflict of interest.
